# Vitamin D status and dental caries in healthy Swedish children

**DOI:** 10.1186/s12937-018-0318-1

**Published:** 2018-01-16

**Authors:** Johanna Gyll, Karin Ridell, Inger Öhlund, Pia Karlsland Åkeson, Ingegerd Johansson, Pernilla Lif Holgerson

**Affiliations:** 1 Department of Odontology, Section of Paediatric Dentistry, Faculty of Medicine, Umeå University, 90185 Umeå, Sweden; 2 Department of Paediatric Dentistry, Faculty of Odontology, Malmö University, Malmö, Sweden; 3 Department of Clinical Sciences/Section of Paediatric Medicine, Faculty of Medicine, Umeå University, Umeå, Sweden; 4 Department of Clinical Sciences, Pediatrics, Lund University, Lund, Malmö, Sweden; 5 Department of Odontology/Section of Cariology, Faculty of Medicine, Umeå University, Umeå, Sweden

**Keywords:** Vitamin D − Children − caries − enamel defects − LL37

## Abstract

**Background:**

Vitamin D is crucial for mineralized tissue formation and immunological functions. The purpose of this study was to evaluate the association between vitamin D status and dental status in healthy children with vitamin D supplementation in infancy and at 6 years of age.

**Method:**

Eight-year-old children who had participated in a vitamin D intervention project when they were 6 years old were invited to participate in a dental follow-up study. They had fair or darker skin complexion and represented two geographically distant parts of Sweden. 25-hydroxy vitamin D in serum had been measured at 6 years of age and after a 3-month intervention with 25, 10 or 2 (placebo) μg of vitamin D_3_ per day. Two years later, caries and enamel defects were scored, self-reported information on e.g., oral behavior, dietary habits and intake of vitamin D supplements was collected, and innate immunity peptide LL37 levels in saliva and cariogenic mutant streptococci in tooth biofilm were analyzed. The outcome variables were caries and tooth enamel defects.

**Results:**

Dental status was evaluated in 85 of the 206 children in the basic intervention study. Low vitamin D levels were found in 28% at baseline compared to 11% after the intervention, and 34% reported continued intake of vitamin D supplements. Logistic regression supported a weak inverse association between vitamin D status at 6 years of age and caries 2 years later (odds ratio 0.96; *p* = 0.024) with minor attenuation after an adjustment for potential confounders. Multivariate projection regression confirmed that insufficient vitamin D levels correlated with caries and higher vitamin D levels correlated with being caries-free. Vitamin D status at 6 years of age was unrelated to enamel defects but was positively associated with saliva LL37 levels.

**Conclusion:**

An association between vitamin D status and caries was supported, but it was not completely consistent. Vitamin D status at 6 years of age was unrelated to enamel defects but was positively associated with LL37 expression.

**Trial registration:**

The basic intervention study was registered at ClinicalTrials.gov with register number NCT01741324 (www.clinicaltrials.gov/ct2/show/NCT02347293) on November 26, 2012.

## Background

Vitamin D is associated with a broad spectrum of biological functions owing to its endocrine, autocrine and paracrine activities [1]. Its reported functions include the regulation of calcium and phosphate metabolism and their deposition in mineralized tissues [2, 3], effects on innate immunity effectors [4], involvement in cognitive functions, roles in blood pressure maintenance and effects related to health outcomes (cardiometabolic conditions, total mortality and aging) [5, 6]. Children and adolescents are particularly vulnerable to the clinical manifestations of insufficient vitamin D because of its central role in bone and tooth formation [3].

Generally, serum 25-hydroxy vitamin D [S-(25(OH)D] levels below <30 nmol/L are considered deficient, 50 nmol/L are insufficient, and >75 nmol/L are suggested as optimal for health [7–9]. According to epidemiological studies, insufficient levels of vitamin D are common in children and adolescents [10] with a higher prevalence reported in areas with less sunshine and in populations with protection against sun exposure or with dark skin complexions [11, 12]. 25-hydroxyvitamin D status is determined by measuring its circulating forms in serum, including the D_2_ and D_3_ variants [2, 7]. Most foods contain relatively small amounts of vitamin *D. major* natural sources include oily fish and eggs, which contain D_3_, and many countries, including Sweden, fortify table spreads and milk with vitamin D_3._

The function of vitamin D in tooth development implies that impaired tooth composition is more prevalent in subjects with vitamin D deficiency [13], but the association may be overlooked since clinical manifestations appear after a significant delay [10, 14]. Based on the effects of vitamin D on tooth quality and the innate immune system, including the defensins and cathelicidins (LL37) [4], studies have evaluated the association between vitamin D levels and dental caries; however, the results are conflicting. Thus, according to some studies, low vitamin D levels/intakes are associated with higher caries prevalence [14–16], but other studies have not observed an association [17]. A recent systematic review of clinical trials assessing the effect of vitamin D on the prevention of dental caries yielded a weak positive effect of vitamin D supplementation with no clear difference in the positive effect between supplementation route, i.e., vitamin D_2_, vitamin D_3_, or ultraviolet radiation [18]. Other studies have assessed associations between vitamin D receptor (VDR) polymorphisms or a combined genetic risk score and dental caries, but these studies have also produced conflicting results [15, 19, 20]. Therefore, further clarification is needed to determine the reasons for the conflicting results, such as studies targeting defined populations and careful monitoring of confounding factors and sufficient variations in vitamin D status.

The primary aim of the present study was to evaluate the association between the vitamin D status of 6-year-old children and their caries status 2 years later. A secondary goal was to evaluate associations of vitamin D status with tooth enamel disturbances and levels of the innate immunity peptide LL37.

## Methods

### Ethical approval

The basic intervention study (DViSUM) was registered at ClinicalTrials.gov (NCT01741324) and approved by the Regional Ethical Review board at Umeå University (Reference: 2012–158-31 M). An addendum was approved (Reference: 2014–103-32 M) for the present dental follow-up study. Data were collected and analyzed according to the guidelines of the Declaration of Helsinki (including written consent from caretakers and children for participation), the Swedish Law on personal data act (PuL) and Law on biobanking, and the guidelines of the Swedish Data Inspection Board.

### Subjects

The present study recruited 8-year-old children who had participated in an intervention study on milk-based vitamin D supplementation at 6 years of age (DViSUM) [21, 22]. DViSUM included 206 children who were proportionally distributed across different living regions [northern Sweden (Umeå, 63°N; *n* = 85) and southern Sweden (Malmö, 55°N; *n* = 121)] and were selected to represent both fair (*n* = 108) and darker skin (*n* = 98) complexions. To be included, the children had to be regular milk consumers. Baseline examinations in DViSUM occurred in November and December of 2012 and included anthropometric measures, blood sampling and questionnaires on information about diet intake and socio-economic conditions. At the first visit in DViSUM, children were randomly assigned to receive 25, 10 or 2 (placebo group), μg of vitamin D_3_ per day in a milk-based supplement for 3 months. Follow-up blood samples were collected when the 3-month intervention period was completed.

**Fig. 1:**
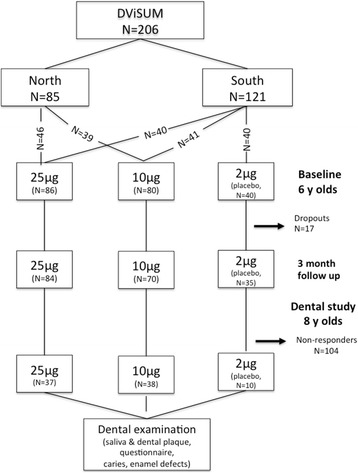
Flow diagram of the numbers of children in the basic intervention DViSUM and placebo groups at baseline, 3 month follow up and at the dental follow up at 8 years of age

Of the 206 children who participated in DViSUM, 85 (41%) consented to participate in an examination of their dental status. Of the 85 children, 37 (42%) in the 25 μg per day group, 38 (45%) in the 10 μg per day group, and 10 (12%) were in the placebo group, and compared to 42%, 39% and 19%, respectively, in the basic study [22]. Dental follow-up occurred in the latter half of 2014. The major reason for non-participation was that the children’s caretakers had moved out of the specified geographic areas. A flow diagram is shown in Fig. 1.

The general characteristics of the study population were low to moderate caries activity and organized dental care from 2 to 3 years of age, including compulsory caries prevention programs. Furthermore, supplementation with vitamin D3 drops (10 μg/day) was strongly encouraged from birth to 2 years of age and up to 5 years in children with dark skin complexions [23].

### Dental examination and data collection

All children were examined by an experienced dentist (JG or KR) for dental caries and enamel defects. At this visit, a questionnaire on tooth brushing and other dental health-related behaviors, including diet with focus on sugar and vitamin D containing foods/food aggregates, use of fluoride and supplements, and health status, medication, and socio-economic information, was administered. All examinations were performed in well-equipped dental offices with good lighting conditions. Caries status was determined by the surface-related decayed-missing-filled index [24], but the missing component was not included since teeth were not lost because of caries. Initial caries and fissure sealants were not included in the decayed and filling components, respectively. Therefore, the permanent dentition D_3-4_FS and the primary dentition d_3-4_ fs were scored (scores 3–4 represent caries lesions into the dentine). The combined measures of D_3-4_FS and d_3-4_ fs are hereafter referred to as dfs/DFS. Bitewing radiographs were captured for special indications, such as when approximal tooth surfaces were unavailable for visual inspection.

Enamel defects were documented and evaluated from photos captured during the clinical examination using criteria defined by the Commission on Oral Health, Research & Epidemiology [25]. Enamel defects, i.e., opacities and signs of enamel hypoplasia, were scored on a 6-level scale, where a score of 0 represents sound enamel and scores of 1–6 represent increasing severity of opacities and hypoplasia. The number of permanent first molars and central upper and lower incisors with a score of 0 or ≥1 were registered.

The scoring of enamel defects was trained and calibrated among two evaluating dentists (JG and KR) and a senior consultant in pediatric dentistry (PLH). Training was conducted with intra-oral photos from anonymous, non-study patients with various manifestations of enamel defects. Diverging scores were discussed to reach a consensus. Reproducibility rates were calculated for the occurrence of enamel defects. The intra-examiner weighted kappa-values (κ) ranged between 0.96 and 0.98. Inter-examiner agreement for the scoring of enamel defects was evaluated using photographs from 10 randomly selected participating children with a κ-value of 0.81.

### Tooth biofilm and saliva sampling and analyses

Tooth biofilm was collected for PCR detection of the caries associated *Streptococcus mutans* and *Streptococcus sobrinus.* Samples were collected from all available supragingival tooth surfaces using sterilized toothpicks, pooled for each participant and stored in TE buffer (10 mM Tris and 1 mM EDTA, pH 7.6) at −80 °C. Genomic DNA was extracted and quality controlled as previously described [26]. The presence of *S. mutans* and *S. sobrinus* was detected using SmF5 and SmR4 primers for *S. mutans* and SsF3 and SsR1 primers for *S. sobrinus* [27].

Paraffin chewing-stimulated whole saliva (3 mL) was collected into ice-chilled test tubes for the analysis of LL37 levels. Samples were aliquoted and stored at −80 °C until further analysis.

### Anthropometric measures

Height and body weight were measured at the baseline DViSUM visit [21, 22], and body mass index, BMI (kg/m^2^), was calculated and converted to a BMI z-score based on WHO reference data for children and adolescents aged 5 to 19 years [28].

### Serum and plasma analyses

Serum 25(OH)D and vitamin D-related components, i.e., calcium, phosphate, magnesium, parathyroid hormone (PTH), alkaline phosphatase (ALP), and osteocalcin in plasma, were analyzed before and after the intervention period as previously described [21, 22]. Briefly, venous blood samples were collected at least 2 h after a meal. The samples were light protected, centrifuged after 30 min, and stored at −20 °C for up to a week and then at -80 °C. S- 25(OH) vitamin D2 and S-25(OH) vitamin D3 levels were analyzed by mass spectrometry on an API 4000 LC/MS/MS system (AB Sciex, Framingham, MA). All other components were analyzed in plasma on Cobas 6000/8000 analyzers (Roche Diagnostics, Mannheim, Germany). Serum levels of 25(OH)D < 50 nmol/L were considered insufficient, and levels <30 nmol/L were considered deficient [29].

### LL37 analysis in saliva

LL37 levels were analyzed using the LL37 Human ELISA kit (HK321, Hycult Biotech, Uden, The Netherlands) according to the manufacturer’s instructions. Briefly, thawed samples were centrifuged, and the supernatant was transferred to antibody-coated microtiter wells to capture LL37, incubated with a biotinylated secondary antibody and detected by streptavidin-peroxidase.

### Data handling and statistical analyses

Continuous, normally distributed variables are presented as the means with 95% confidence limits (CI). Differences between group means were either evaluated with Student’s t-test for comparisons between two groups and ANOVA for more than two groups. Differences in dietary intake were tested with the Mann-Whitney non-parametric test. When appropriate, mean values were standardized for potential confounders, such as living region, skin type, and vitamin D supplement intake, using general linear modeling (glm). Categorical variables are presented as numbers or proportions, and the Chi^2^ test was used to determine differences in distributions. All tests were two-sided, and *p*-values <0.05 were considered statistically significant.

Logistic regression analysis was used to evaluate the adjusted odds ratio (OR) and 95% confidence limits (CI) for caries with vitamin D status as the independent variable and potential confounders included as covariates. Vitamin D was evaluated as a continuous measure. A dichotomous caries classification (caries-free or caries-affected) was used to account for zero-inflated dfs/DFS scores. Three models were evaluated: model 1 (basic model) was adjusted for the number of teeth, tooth brushing (once or twice a day), presence or absence of *S. mutans*, father’s education level (<12 years or ≥12 years of school attendance), and region of residence; model 2 also included BMI (z-scores) and intake of a vitamin D supplement at the time of the dental examination; and model 3 also included skin type. Since all blood samples were collected in the same season, adjustment for season was not needed. Further adjustments, including the parents’ country of origin and reported intake of various sweet products, were tested, but they did not affect the results. Firth’s penalized likelihood approach was used to address the small group sizes using proc. logistic in SAS 9.4 (SAS Institute Inc., Cary, NC, USA) and odds ratios with and without correction are presented.

Multivariate principal component analysis (PCA) and partial least square (PLS) regression analyses were used to search for clustering of subjects and variables associated with caries (yes/no) and number of teeth with defective enamel, respectively. Following a screening step in which variables that were influential in explaining the variation in the dependent variables, i.e., Variable Importance in Projection (VIP) values >1 among all serum/plasma components, anthropometric measures, diet, oral health behavior, socio-economic, and medical variables (*n* = 106) were identified. These variables were then entered into final PCA/PLS models for caries and enamel defects.

Analyses were performed using SPSS version 23 (IBM Corporation, Armonk, NY, USA). SIMCA P+ version 12.0 (Umetrics AB, Umeå, Sweden) was used for PCA and PLS modeling.

### Power calculation

Dental caries was the primary outcome measure, and secondary outcomes were enamel defects and LL37 levels in saliva. G*Power (http://www.gpower.hhu.de/) was used to calculate the power to find a statistically significant difference in mean vitamin D levels between children with or without caries given the number and distribution of participants. With 85 participants, α = 0.05, an effect size of 0.6 (difference in group means = 12 and SD = 18 based on the distribution of all serum values), and two-tailed testing, we have 77% power to detect a statistically significant difference.

## Results

### Vitamin D status and other participants’ characteristics

Vitamin D status before and after the intervention in the basic DViSUM cohort (*n* = 206) and the children in the dental subgroup (*n* = 85) are presented in Table 1 together with information on dietary intake. Additional information for the DViSUM basic study is found in Karlsland Åkeson et al. and Öhlund et al. [21, 22]. The overall (n = 206) mean vitamin D level was 55.4 nmol/L (95% CI: 52.8, 58.0) at baseline with a mean increase of 30% in the high and 19% in the low intervention groups and no change in the placebo group (23). This result were in accordance with the levels found in the subgroup employed in the present study, i.e., 60.4 nmol/L (95% CI: 56.4, 64.3) and 76.3 nmol/L (95% CI: 72.2, 80.3) at baseline and follow-up, respectively (Table 1). However, children who participated in the dental follow-up study had 8 nmol/L higher vitamin D levels at baseline and 3-month follow-up than those who did not (both *p* = 0.002). Skin complexion and vitamin D intake from fortified milk and spreads were identified as the major determinants for vitamin D status in the DViSUM cohort [21]. A larger proportion of children who participated in the dental follow-up study had fair skin compared to those who did not (68 and 41%, respectively; *p* = 0.001), but their reported milk and fish intakes were similar (Table 1). Intake of sweet products was not monitored at 6 years of age, but at 8 years of age, intake of several sweet products was common. Mean intake of products with added sucrose was 1.7 times per day (Table 1).

**Table 1:** Vitamin D status and diet intake in 6 year olds in the DViSUM study group (*n* = 206) and in the nested dental study sub-group (*n* = 85). Data are presented as mean with 95% CI. ND = not determined

	DViSUM	Dental study sub-group	
	6 years of age n = 206	6 years of age n = 85	8 years of age n = 85
Vitamin D status, nmol/L			
at baseline	55.4 (52.8, 58.0)	60.4 (56.4, 64.3)	ND
after intervention	71.5 (68.6, 74.3)	76.3 (72.2, 80.3)	ND
Intake of vitamin D foods			
Milk^a^, mL/day	535 (484, 587)	562 (478, 646)	574 (521, 629)
Cheese^b^, g/day	20.0 (13.5, 26.5)	14.7 (9.9, 19.2)	17 (12, 22)
Eggs, g/day	13.4 (8.2, 18.5)	14.4 (12.5, 17.6)	ND
Fatty fish, g/day	14 (11.7, 17.5)	14.2 (12.6, 17.2)	13 (9, 16)
Table spreads, g/day	19.7 (17.9, 21.5)	20.9 (17.9, 23.9)	ND
Vitamin D supplement, % with reported intake	14	10	34
Intake of sweet products			
Sum of sucrose product, frequency/day	ND	ND	1.7 (1.1, 2.3)
Sodas with sucrose, frequency/day	ND	ND	0.4 (0.3, 0.5)
Cookies and sweet buns, frequency/day	ND	ND	0.53 (0.28, 0.79)
Non-sweet snacks, frequency/day^c^	ND	ND	0.15 (0.12, 0.18)
Fruits, frequency/day	ND	ND	1.5 (1.3, 1.7)

^a^including non-fermented milk, sour milk, and yoghurt (natural and sweetened)

^b^including cheese, and cottage cheese

^c^including crisps, cheese doodles, popcorn

Similar to in the basic DViSUM cohort [21, 22], serum 25 (OH)D levels varied by skin type and region of residence in the dental subgroup. Thus, children with darker skin (*n* = 27) had significantly lower 25 (OH)D levels than children with fair skin (*n* = 58) (mean (95% CI): 49.4 (42.0, 56.8) and 65.4 (61.3, 69.6) nmol/L, respectively, *p* < 0.001). In the former group, 59.3% had insufficient levels compared with 13.8% in the latter group (p < 0.001). Further, children in southern Sweden had significantly lower S-25 (OH)D levels than children in northern Sweden (mean (95% CI): 54.7 (48.9, 60.4) and 64.9 (59.7, 70.1), respectively; *p* = 0.009). Insufficient S-25 (OH)D levels were seen in 42.1% in the former compared to 17.0% in the latter group (*p* = 0.011).

### Vitamin D and caries status

Caries-affected children had a mean of 4.5 decayed or filled tooth surfaces (dfs/DFS) (95% CI: 3.3, 5.8) at 8 years of age (Table 2). In univariate analyses, vitamin D levels did not differ between children with or without caries, although the proportion with <50 nmol/L of S-25[OH]D tended to be higher among children with caries (Table 2). dfs/DFS scores did not differ significantly between the three vitamin D intervention groups [2.5 (0, 6.3), 2.1 (1.0, 3.1) and 1.7 (0.7, 2.7) for 2, 10 and 25 μg, respectively; *p* = 0.758]. Children with <50 nmol/l of S-25[OH]D after the intervention had a mean dfs/DFS of 5.8 compared to 1.4 for those with >50 nmol/L (*p* = 0.001). The trend was similar for the baseline S-25[OH]D strata (2.9 versus 1.6: *p* = 0.086). A darker skin complexion was significantly more common among those with caries than without (*p* = 0.014, Table 2). No association was seen between caries status and reported intake frequency of any sweet or other food item assessed in the questionnaire (data not shown). The proportions reporting vitamin D supplement intake did not differ between caries groups (Table 2) or vitamin D intervention groups (data not shown), but it was more common among children who had an insufficient vitamin D status or not at baseline (58% and 25%, *p* = 0.004) and after the intervention (78% and 30%, *p* = 0.005). Notably, after the second blood analyses, parents were informed about the child’s vitamin D status and supplements were recommended for those with low levels. This recommendation was reflected in that at 8 years of age, 34% reported intake of a vitamin D supplement compared to 14% before the intervention started (Table 1).

**Table 2:** Characteristics of dental study group participants by caries status. Data are presented as mean with 95% CI for continuous measures and proportion (%) for categorical measures. Differences between groups were tested with Students t-test and Chi-square test, respectively

	Caries-free *n* = 48	Caries *n* = 37	p-value
Baseline			
Age years, mean (95% CI)	6.4 (6.2, 6.6)	6.3 (6.0, 6.5)	0.259
Boys, %	52.1	32.4	0.070
Region, south; north, %	52.1; 47.9	35.1; 64.9	0.119
Fair; darker skin, %	79.2; 20.8	54.1; 45.9	0.014
Mother education, % ≥12 years	56.3	51.4	0.653
Father education, % ≥12 years	68.1	51.4	0.126
BMI, z-score	0.23 (−0.06, 0.52)	0.29 (−0.03, 0.62)	0.764
Vitamin D supplement, % with reported intake	31.3	38.9	0.466
Insufficient vitamin D status, %	20.8	37.8	0.084
S-25(OH) D, nmol/L	62.6 (57.3, 67.8)	57.4 (51.3, 63.5)	0.195
S-Calcium, mmol/L	2.43 (2.41, 2.45)	2.45 (2.43, 2.47)	0.339
S-Phosphate, mmol/L	1.57 (1.53, 1.61)	1.51 (1.46, 1.55)	0.033
S-Magnesium, mmol/L	0.88 (0.86, 0.89)	0.85 (0.84, 0.87)	0.015
Parathyroid hormone (PTH), pmol/L	3.86 (3.47, 4.30)	3.68 (3.32, 4.04)	0.512
Osteocalcin, μg/L	86.2 (79.1, 93.4)	81.3 (73.1, 89.6)	0.364
Alkaline phosphatase, μkat/L	3.94 (3.71, 4.16)	3.99 (3.62, 4.35)	0.812
Dental examinations			
Age, years	8.3 (8.1, 8.5)	8.1 (7.8, 8.3)	0.258
Number of teeth			
total number	23.5 (23.2, 23.7)	22.9 (22.4, 23.5)	0.059
deciduous teeth	11.5 (10.7, 12.3)	11.5 (10.6, 12.5)	0.968
permanent teeth	12.0 (11.2, 12.7)	11.4 (10.5, 12.4)	0.368
Caries status score (dfs/DFS)	0	4.5 (3.3, 5.8)	<0.001
Enamel defects			
single tooth, %	62.5	63.9	0.896
multiple teeth, %	45.8	44.4	0.899
Tooth brushing twice a day, %	88.9	94.3	0.397
Saliva analyses			
LL37, ng/mL	1.01 (0.74, 1.29)	1.56 (1.24, 1.88)	0.012
*S. mutans,* % positive by PCR	27.1	33.3	0.535
*S. sobrinus* % positive by PCR	0	0	—

As a next step, we evaluated the risk of developing caries according to vitamin D levels (continuous measure) at baseline and after the 3-month intervention using a logistic regression model that included potential confounders. In the basic model (model 1), higher baseline vitamin D levels were significantly associated with less caries [OR (95% CI) 0.961 (0.929, 0.995; *p* = 0.024)] which remained after Firth’s correction (Table 3). Additional adjustments for BMI and reported intake of vitamin D supplement at caries examination attenuated the results slightly (0.967 (0.931, 1.005; *p* = 0.085); Table 3) and they were no longer statistically significant after Firth’s correction. The results were similar when vitamin D levels at follow-up were used as the independent variable (Table 3). Backward elimination indicated that S-25[OH]D levels at baseline, tooth brushing, number of teeth, and living region were independently associated with having caries (*p* = 0.01, Table 3).

**Table 3:** Odds ratios to have dental caries or not by increasing vitamin D status. Serum vitamin D levels are at 6 years of age and caries status at 8 years of age

	Logistic regression					
	Baseline			3 months after intervention		
	Odds ratio	95% CI	p-value	Odds ratio	95% CI	p-value
Method: Variables entered						
Model 1^a^	0.961	0.929, 0.995	0.024	0.966	0.936, 0.997	0.030
Model 2^b^	0.962	0.928, 0.998	0.037	0.971	0.939, 1.003	0.075
Model 3^c^	0.967	0.931, 1.005	0.085	0.975	0.943, 1.009	0.148
With Firth’s correction						
Model 1^a^	0.966	0.935, 0.998	0.040	0.969	0.940, 0.999	0.044
Model 2^b^	0.970	0.935, 1.008	0.117	0.975	0.943, 1.008	0.134
Model 3^c^	0.973	0.936, 1.010	0.153	0.979	0.947, 1.013	0.232
Method: Backward elimination (variables retained in final model)						
D vitamin status	0.958	0.926, 0.990	0.01	0.971	0.940, 1.003	0.08
Number of teeth	0.666	0.412, 1.061	0.087	0.598,	0.366, 0.975	0.039
Living region^d^	3.13	1.05, 9.33	0.040	2.62	0.842, 8.16	0.096
Tooth brushing^d^	0.099	0.008, 1.16	0.065	Not retained		
Skin type^d^	Not retained			3.08	1.02, 9.35	0.047

^a^Model 1 with caries (yes/no) as dependent variables and serum levels of vitamin D, number of teeth, tooth brushing, *S. mutans*, parental education, and living region as covariates

^b^model 1+ BMI, and reported intake of vitamin D supplement at the caries examination

^c^model 2 + skin type

^d^The reference categories were southern Sweden (living region), brushing <twice a day (tooth brushing), and fair skin (skin type)

**Fig. 2:**
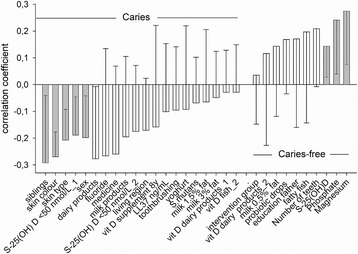
PLS correlation coefficients from multivariate modelling with caries status (yes/no) as dependent variables. Correlation coefficients with 95% CI for the variables in the final mode are presented. Bars for which the 95% whisker do not pass zero are statistically significant

Finally, PLS modeling was applied with caries status (yes/no) as dependent variables and variables with a screening VIP value > 1 in the independent block. PLS identified one significant component with an explanatory (R^2^) and predictive (Q^2^) capacity of 29.8% and 12.1%, respectively. Having caries was significantly associated with number of siblings, skin color and type, and having less than 50 nmol/l of 25(OH)D levels in serum (Fig. 2). Being caries-free was significantly associated with higher levels of magnesium, phosphate and 25(OH)D in serum (Fig. 2).

### Vitamin D status and enamel defects

The prevalence of enamel defects on the permanent first molars and central upper and lower incisors did not differ between children with insufficient and sufficient vitamin D levels at 6 years of age. Six children had serum vitamin D levels corresponding to a vitamin D deficiency, i.e., <30 nmol/L, and 4 of these children had multiple enamel defects on the assessed teeth, but 2 did not. Thus, in this population, baseline levels of S-25 (OH)D or any of the other serum components did not differ between the children with or without enamel defects on the permanent first molars and central upper and lower incisors (data not shown).

PLS modeling only identified intake of a vitamin D supplement at 8 years of age as associated with having defects, which likely reflects reversed causality.

### Vitamin D status and LL37 levels in saliva

LL37 is an innate immunity peptide for which expression has been linked to vitamin D status [4]. In the dental study group, region-adjusted mean LL37 levels were lower in children with insufficient serum vitamin D status than in children with serum 25(OH)D levels ≥50 nmol/L after the 3-month intervention [1.09 (0.87, 1.30)] and [2.38 (1.77, 2.99), respectively; *p* < 0.001]. No difference was observed in LL37 levels between children who reported vitamin D supplement intake at 8 years of age or not. The mean LL37 levels in saliva (adjusted for region of residence) were higher among children who had caries at 8 years of age.

## Discussion

The primary goal of the present study was to evaluate the association between vitamin D status and prospective caries status in children selected from a population with overall low caries prevalence, high milk consumption and vitamin D supplementation in infancy [21, 30]. The results support an inverse association between vitamin D status and caries, i.e., higher vitamin D and less caries, although the results were not fully consistent. No association was found with enamel defects.

Most studies evaluating the association between vitamin D status and dental caries use a cross-sectional design [14, 31]. Caries development is normally a slow process with several years of delay before a cavity is observed. Therefore, the serum 25(OH)D level at the time of caries scoring may or may not be representative of the period when caries symptoms developed [14]. Therefore, we examined the participants’ caries status 2 years after vitamin D levels were assessed. Although the participants’ vitamin D status was not followed during the two years between the initial vitamin D measurement and dental examination, information on vitamin D supplement intake at the time of the dental examination was available. The children in the present study were nested in a study in which vitamin D status was analyzed before and after a 3-month treatment with milk-based supplements with different vitamin D concentrations at 6 years of age. When the follow-up blood samples were analyzed, parents of children with serum levels <50 nmol/L were advised to administer a vitamin D supplement to their children. We have no information on individual compliance with this advice, but at the time of dental examination, 78% of children with <50 nmol/L (insufficient vitamin D status) in their second blood sample reported that the child was given a vitamin D supplement. Therefore, one might hypothesize that the serum 25(OH)D levels measured after the intervention would better reflect the levels over the 2-year interval for most study subjects. On the other hand, the results from the models employing the baseline values may better reflect habitual levels and the situation during the period between 2 and 6 years of age when teeth mineralized and caries development likely began. However, to be able to assess the associations properly, the question should be addressed in a longitudinal study with repeated measures of both serum 25(OH)D levels and caries status from early childhood to the age of caries scoring and should include children with more selective diets. In the present study, consuming milk was an inclusion criterion.

The saliva LL37 concentration was lower in children who had an insufficient D vitamin levels than in children who had sufficient serum levels at follow-up. This result was stable in sensitivity analyses stratified by supplement intake at 8 years of age. LL37, a 37-amino acid peptide generated from proteolytic cleavage of the extracellular domain of the 18-kDa hCAP18 protein from epithelial cells and neutrophils [32, 33], is the only cathelicidin-derived antibacterial peptide in humans. Vitamin D has been shown to have a specific role in the expression of the cathelicidins, and several studies have confirmed the association between vitamin D and LL37 levels [4]. LL37 has been linked to several biological innate immune functions, including those in periodontal disease and psoriasis [34, 35]. LL37 has also been implicated in the oral microbiota, but the results from studies on its association with dental caries are inconsistent [36, 37]. In the present study, children with caries had higher LL37 levels, which may indicate oral microbiota-induced expression. Although these findings are consistent with some previous publications [38, 39], the results should be interpreted with caution and evaluated in a larger study in which vitamin D status is measured closer to the time of LL37 analysis.

According to previous studies, enamel defects in the form of opacities and hypomineralization are more prevalent in patients with vitamin D deficiency [40]. In the present population, the prevalence of enamel defects on permanent first molars or central incisors did not differ between children with serum levels <50 nmol/L at 6 years of age and children with sufficient vitamin D at this age. This finding was expected since the evaluated teeth were mineralized during the first years of life, and all parents in Sweden are encouraged to provide vitamin D_3_ supplements of 10 μg/day in drop form to their children from birth to 2 years of age. The drops are available free of charge. Children with dark skin, or those with little outdoor activity, and children who do not receive fortified products or eat fish are advised to continue taking the vitamin D drops up to 5 years of age [29, 41]. Therefore, during these early years, intake from supplements rather than UV light exposure is likely to be the major determinant of vitamin D status. This hypothesis was also supported by the finding that the prevalence of enamel defects on the specified teeth did not differ between children with fair or dark skin in the present study, even though their vitamin D status differed at 6 years of age.

The strengths of the present study include that vitamin D status was measured and not based on self-reported intake only, that caries were evaluated prospectively and were scored by experienced pediatric dentists who took X-rays when approximal surfaces were not accessible and that the study group was very well characterized. However, there are limitations that should be acknowledged when the results are interpreted. The study group is small with limitations in stratification, vitamin D status was not reanalyzed later than the intervention follow-up, and there is a potential risk of response bias in the questionnaire replies. It should also be acknowledged that the study group represents populations characterized by vitamin D supplementation in the first living years and regular dental care.

## Conclusions

Based on the present results, we conclude that the results from Schroth et al. [14] of an inverse association between vitamin D status and caries were supported. However, the comparably small study group and weak association with attenuation from confounder adjustment suggest the need for replication. Vitamin D status was unrelated to enamel defects on permanent incisors and molars in the present circumstances, whereas an association between vitamin D status and LL37 levels was supported.

## Abbreviations

ALP: Alkaline Phosphatase
BMI: Body Mass Index
CI: Confidence Interval limit
DFS: Decayed Filled Surface
GLM: General Linear Modelling
OR: Odds ratio
PCA: Principal Component Analysis
PCR: Polymerase Chain Reaction
PLS: Partial Least Square Regression analysis
PTH: ParaThyroid Hormone
PuL: Swedish law on personal data act
